# OpenStreetMap mapathons support critical data and visual literacy instruction

**DOI:** 10.5195/jmla.2020.1070

**Published:** 2020-10-01

**Authors:** Bethany Sheriese McGowan

**Affiliations:** 1 bmcgowa@purdue.edu, Assistant Professor of Information Studies and Health Sciences Information Specialist, Life Sciences Library, Purdue University, West Lafayette, IN

## Abstract

OpenStreetMap (OSM) mapathons can offer a learner-centered means for critical data literacy and visual literacy instruction. Mapathons are coordinated humanitarian mapping events in which participants use geographic information system (GIS) data and satellite imagery to create open-source maps for humanitarian support. Visual mapping is an effective learning activity because it encourages students to use big data to create a deliverable—an open-source map—that allows instructors to engage learners in data literacy and visual literacy at the highest cognitive level.

OpenStreetMap (OSM) mapathons can offer a learner-centered means for critical data literacy and visual literacy instruction. Mapathons have been used as coordinated humanitarian mapping events, in which participants use geographic information system (GIS) data and satellite imagery to create open-source maps for humanitarian support. They have been used to improve coverage of under-mapped communities and support disaster relief efforts, economic assessments, and energy management analyses.

The Humanitarian OSM Team (HOT) provides free resources and training to prospective mapathon hosts. Instructors seeking hands-on mapping experience can visit the HOT site to practice mapping and to learn to host mapathons. The Missing Maps site allows mapathon hosts to advertise public events and allows mappers to view information about upcoming mapathons. Examples of how libraries have hosted mapathons to support disaster relief efforts have been highlighted in the news, showcasing the work of librarians at MIT and at Columbia University.

The *Association of College & Research Libraries (ACRL) Visual Literacy Competency Standards for Higher Education* can help guide the design of cocurricular and extracurricular instruction that teaches students to critically view, use, and produce maps, and help develop appropriate learning objectives, activities, and assessments. Learning objectives for mapathons can center on one or more of the competencies established for a visually literate individual in higher education, especially understanding the “ethical, legal, social, and economic issues surrounding the creation and use of images and visual media” [1]. *Visual Literacy for Libraries: A Practical, Standards-Based Guide*, a resource that includes worksheets and activities that support discussions on the ethical and social aspects of map creation and trustworthiness, further supports visual literacy instruction [2].

At Purdue, these ACRL standards and resources were used to develop data literacy instruction, and students were encouraged to consider using information from these sources to assess the quality of their map data. From one to three extracurricular mapathons have been successfully hosted each semester since fall 2017, mostly for undergraduate students in health sciences–centered and data sciences–centered learning communities. Learning communities have proved to be an effective teaching environment for the mapathons because most learning communities require student participation in extracurricular activities like mapathons, which offer synergistic opportunities for librarians and learning community stakeholders.

Venues have included library spaces, computer labs, and active-learning classrooms, as well as lounge and study spaces in dorms. Mapathons can also be hosted completely online. Mapathon events have ranged in size from as few as six participants to as many as sixty participants. For example, the Purdue University Libraries 2017 GIS Day mapathon gained international participation after being highlighted on a popular blog for mappers. Alternatively, mapathons have been completed in fifty-minute sessions if students complete an hour of mapping prework, making them useful as cocurricular instruction methods in courses.

Visual mapping is an effective learning activity because it encourages students to use big data to create a deliverable—an open-source map—that allows instructors to engage learners in data literacy and visual literacy at the highest cognitive level. Once students create an OSM account, they can map during mapathons or individually on their own time. Students learn to validate data sources, observe and question data gathering methods, and question data quality—practices that improve both critical data literacy and evidence-based decision making.

Maps can also be used as evidence to formulate and support research inquiries. For example, what can a map tell you about community health needs? What challenges might a community face if the closest freshwater source is 100 miles away or if the community is disconnected from major road infrastructure? Figure 1 illustrates regional map data in OSM that provides information about a community's water features, power features, traffic roads, pedestrian and bike paths, and points of interest, including markets, restaurants, and more.

**Figure 1:**
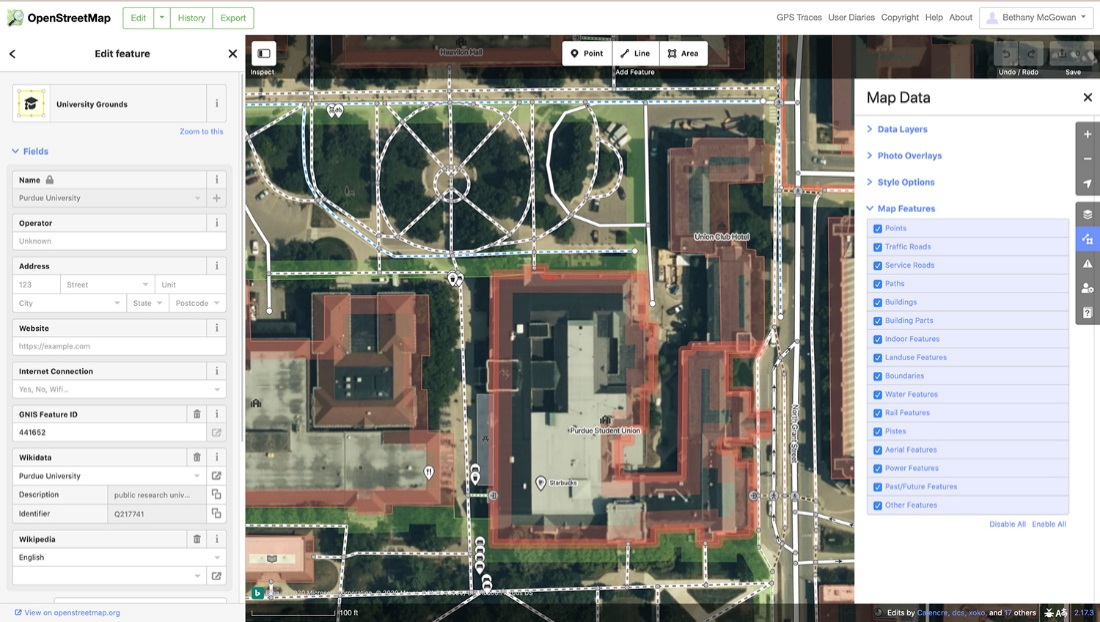
An image of an OpenStreetMap in Edit mode
